# Tongue-driven sonar beam steering by a lingual-echolocating fruit bat

**DOI:** 10.1371/journal.pbio.2003148

**Published:** 2017-12-15

**Authors:** Wu-Jung Lee, Benjamin Falk, Chen Chiu, Anand Krishnan, Jessica H. Arbour, Cynthia F. Moss

**Affiliations:** 1 Applied Physics Laboratory, University of Washington, Seattle, Washington, United States of America; 2 Department of Psychological and Brain Sciences, Johns Hopkins University, Baltimore, Maryland, United States of America; 3 Indian Institute of Science Education and Research (IISER) Pune, Pune, Maharashtra, India; 4 Department of Biology, University of Washington, Seattle, Washington, United States of America; Queen Mary University of London, United Kingdom of Great Britain and Northern Ireland

## Abstract

Animals enhance sensory acquisition from a specific direction by movements of head, ears, or eyes. As active sensing animals, echolocating bats also aim their directional sonar beam to selectively “illuminate” a confined volume of space, facilitating efficient information processing by reducing echo interference and clutter. Such sonar beam control is generally achieved by head movements or shape changes of the sound-emitting mouth or nose. However, lingual-echolocating Egyptian fruit bats, *Rousettus aegyptiacus*, which produce sound by clicking their tongue, can dramatically change beam direction at very short temporal intervals *without* visible morphological changes. The mechanism supporting this capability has remained a mystery. Here, we measured signals from free-flying Egyptian fruit bats and discovered a systematic angular sweep of beam focus across increasing frequency. This unusual signal structure has not been observed in other animals and cannot be explained by the conventional and widely-used “piston model” that describes the emission pattern of other bat species. Through modeling, we show that the observed beam features can be captured by an array of tongue-driven sound sources located along the side of the mouth, and that the sonar beam direction can be steered parsimoniously by inducing changes to the pattern of phase differences through moving tongue location. The effects are broadly similar to those found in a phased array—an engineering design widely found in human-made sonar systems that enables beam direction changes *without* changes in the physical transducer assembly. Our study reveals an intriguing parallel between biology and human engineering in solving problems in fundamentally similar ways.

## Author summary

It is well known that animals move their eyes, ears, and heads towards stimuli of interest to selectively gather information in complex environments. Interestingly, lingual-echolocating fruit bats, which generate sonar signals for object localization by clicking their tongues, can rapidly switch the direction of the sonar beam without changing head aim or mouth shape. The mechanism underlying this capability has intrigued scientists and engineers alike. In this study, we used a combination of experimental measurements and theoretical modeling to solve this mystery. We discovered that the focus of this bat’s sound beam shifts systematically across a range of angles as the sonar frequency increases. This unusual multi-frequency structure can be captured by modeling the sound emission as an array of sound sources located along the side of the mouth and driven by the clicking tongue. Changing only the position of the tongue in this model can steer the sonar beam in different directions, showing an effect broadly similar to that found in a human-made sonar phased array—a design that enables changing beam direction without changing the physical transducer assembly. Our study thus reveals an intriguing parallel between biology and human engineering, which arrived at fundamentally similar solutions to the same problem.

## Introduction

Adaptive sampling is a universal principle of animal sensing that has recently gained popularity in engineering practices [1]. By selectively gathering information pertinent to behavioral goals, animals are able to efficiently parse and process information in complex environments [2,3]. Echolocating animals, such as bats and toothed whales, emit sounds and analyze returning echoes to build representations of their surroundings for navigation and foraging [4,5]. Their sound beams are highly directional and function as an “acoustic flashlight” to enhance information intake from the illuminated space while suppressing clutter in complex environments [6,7]. Importantly, the narrow sampling volume of a highly directional beam makes it crucial to adaptively adjust the beam direction to build a comprehensive acoustic image of the environment [8]. This is analogous to the process of consecutive foveal fixations and saccadic eye movements to scan a visual scene during search and reading [9].

Just as movements of head, eyes, and ears are essential in adaptive acquisition of sensory information [9,10], an important observation from previous studies is that adaptive changes in directional biosonar sampling are enabled by physical movements of sound-emitting structures. Among the bat species that produce echolocation signals using their larynx (laryngeal echolocators), beam control is achieved by either turning the head and changing the mouth gape for oral-emitting bats [11] or deforming specialized facial appendages (noseleaves) for nasal-emitting bats [12–14]. However, this empirical principle of sonar beam control does not apply to lingual-echolocating species that produce echolocation signals with their tongue, such as the Egyptian fruit bat (*R. aegyptiacus*).

The Egyptian fruit bat emits alternating left- and right-pointing broadband “clicks” in pairs, rather than singly, unlike most bat species [15,16]. An early report suggested that each pair of clicks are produced by a series of complex movements, as the bat flicks the tongue away from the bottom of the mouth [17]. The paired clicks point in directions as large as 60° apart, but are separated in time by only approximately 20 msec. In addition, the inter-click angle within each pair of clicks is adaptively controlled for accurate target localization [15,18]. Curiously, no clear head or mouth movements have been observed between the emission of these clicks. How, then, do these bats achieve such efficient sonar beam direction steering? We hypothesize that these capabilities are directly linked to the bat’s tongue-driven click production mechanism. Specifically, we hypothesize that the tongue motion during click generation results in rapid alternation of click directions within each pair, and that the sonar beam aim is adjusted by changing the relative position of the clicking tongue with respect to the emission aperture (the narrowly-parted lips), which creates variation of phase differences across the aperture. We predict that these properties will result in measurable features in the bat’s broadband sonar beam pattern, and that the observed beam pattern features can be captured by a theoretical model. Similar mechanisms have been suggested for horizontal beam steering by nose-emitting laryngeal-echolocating bats [19,20]. However, there has been no experimental evidence to validate this possibility in either laryngeal- or lingual-echolocating bats. In this study, we test these predictions by combining broadband acoustic measurements from flying bats with a numerical model of biosonar beam formation to obtain insight into the mechanisms shaping sonar beam directionality of click pairs produced by the Egyptian fruit bat.

## Results and discussion

**Fig 1:**
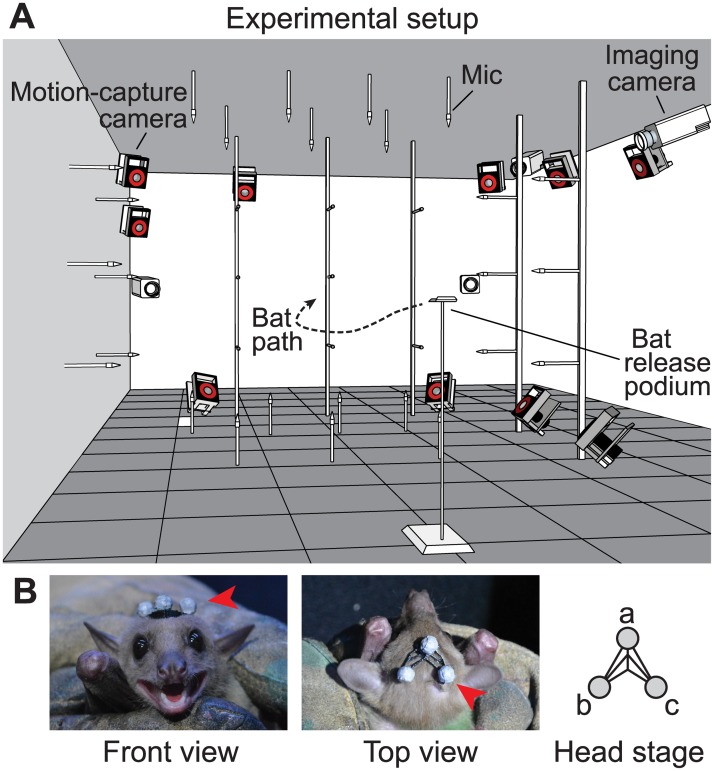
Setup of the experiment. (A) Experimental space and arrangement of instruments, including a three-dimensional array of 34 ultrasonic microphones, 10 high-speed motion-capture cameras, and 4 high-speed imaging cameras. (B) The head stage (indicated by red arrows) used to track the bat’s head movements.

**Fig 2:**
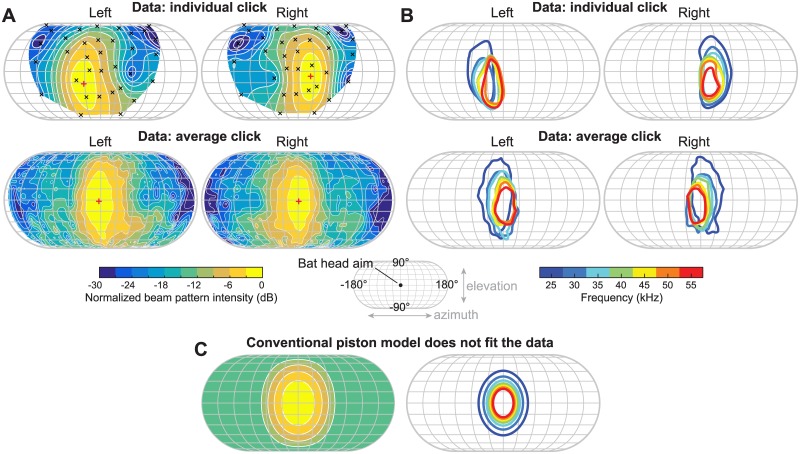
Measured beam pattern features of *R. aegyptiacus*. (A) Normalized beam pattern at 35 kHz for a pair of consecutive clicks (top) and the average clicks obtained by merging all measured clicks (bottom). Crosses (“x”) indicate projected microphone locations, and plus signs (“+”) indicate the locations of sonar beam center. (B) Multi-frequency beam pattern structure of the same pair of clicks shown in (A) (top) and of the average clicks (bottom). The −3 dB contours (main lobe locations) of normalized beam pattern across multiple frequencies are color-coded. The average clicks appear more centered in both (A) and (B) because the 35-kHz beam axis of each individual click was aligned to the origin before averaging. (C) Model predictions of beam pattern and multi-frequency structure of a circular piston, plotted in the same color scales as in (A) and (B). The conventional piston model does not capture the beam pattern features of *R*. *aegyptiacus*. Note that all beam patterns in this study are plotted using the Eckert IV map projection from the bat’s perspective (see middle inset for orientation). The “stretch” in elevation of this map projection was compensated for in all the analyses.

We reconstructed the biosonar beam pattern of free-flying *R*. *aegyptiacus* across the entire azimuth-elevation domain using a three-dimensional ultrasonic microphone array and synchronized high-speed video recordings (Fig 1 and S1 Video). The beam patterns of these broadband sonar clicks are significantly elongated in elevation (Fig 2A, S1 Fig), with mean azimuthal and elevational −3 dB beam widths of 25.2° and 36.6°, respectively, and a mean aspect ratio of 1.49 (characterized by the −3 dB best-fitting ellipse at 35 kHz, see Materials and methods). The beam patterns also contain uneven azimuthal intensity distribution across all frequencies (Fig 2A and S1 Fig), corroborating with previous results from narrowband measurements [15]. In addition, the sonar beams exhibit an unusual multi-frequency structure (Fig 2B): the main lobe is directed more laterally at lower frequencies and points more toward the medial axis (directly in front of the animal) at higher frequencies, with the center of the beam shifted by 17.8° and 14.0° from 25 to 55 kHz for the average left and right clicks, respectively. This multi-frequency structure has not been observed in other echolocating animals, including bats and toothed whales. Furthermore, the above features are conserved across all individual left- and right-pointing clicks and the average clicks. Here, the average clicks were constructed to augment individual click measurements, which were sampled more coarsely in space. Results from a series of Monte Carlo simulations suggest that our beam pattern measurements are not affected by potential spatial sampling bias (S1 Text).

The beam pattern features measured in our study cannot be explained by the conventional “piston model,” which models oral-emitting echolocation as a piston-shaped emitter in an infinite baffle [21,22]. The piston model predicts a circularly varying beam intensity distribution with a concentric multi-frequency structure (Fig 2C). For decades, this prediction has been shown to be consistent with the sonar beam patterns of laryngeal-echolocating, oral-emitting bats as well as toothed whales [5,23]. Although slight deviations from the piston model have been observed [24], none exhibits the distinctly oval beam shape and asymmetric frequency-dependent main lobe direction variation shown in our data.

The frequency-dependent main lobe direction variation (Fig 2B) is reminiscent of the features of frequency-scanning phased sonar/radar arrays, whose beams point toward different directions in a frequency-dependent manner [25]. Based on this insight and prior observations of the bat’s click production mechanism [4,17], we investigated whether a beam formation mechanism similar to a phased array might reproduce the observed beam pattern features, and thus explain biosonar beam formation and steering in lingual echolocation.

**Fig 3:**
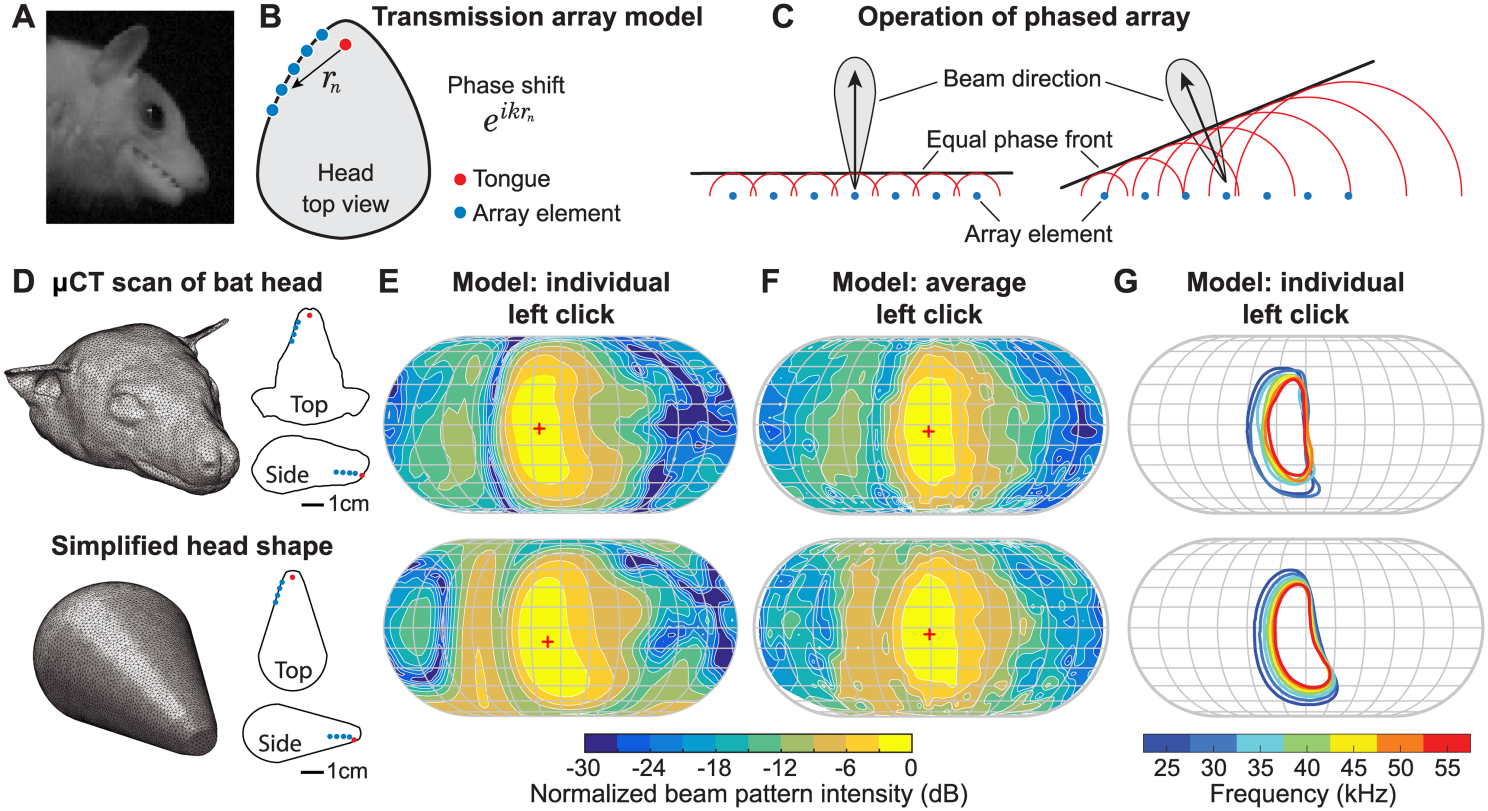
The transmission array beam pattern model. (A) A close-up view of the bat’s mouth during click emission extracted from a high-speed video (S2 Video). (B) Illustration of model formulation. The beam pattern is formed by coherent summation of the contributions of array elements along the narrow mouth opening. The phase of each element is shifted by $e^{ikr_{n}}$, determined by its distance to the clicking tongue (*r*_*n*_) and frequency (through the wavenumber *k*). (C) Operation of an engineered phased array. The sound beam is directed perpendicularly from the array when all elements transmit in phase (left). The beam is steered in other directions when the elements transmit with systematically varying phase shifts (right). Our model differs from the engineered phased array in that the phase shifts at array elements are not individually controlled. Instead, it is the overall phase shift pattern that is varied by the tongue position change. (D) Shape representations of the bat head used in the BEM model implementation. The red and blue dots show the locations of the tongue and array element, respectively. (E and F) Model beam patterns of individual clicks (E) and average clicks (F) at 35 kHz, predicted using the bat head shapes shown in (D). The model average clicks were reconstructed based on Monte Carlo simulated data generated using bat and microphone locations recorded during the experiment (S1 Text). (G) Multi-frequency structure of the individual model clicks shown in (E). The plotting conventions in (E-F) are identical to those in Fig 2. BEM, boundary element method; μCT, micro computed tomography.

The Egyptian fruit bat emits echolocation signals through clenched teeth and lips parted, or occasionally with teeth and lips parted, without rapid modulations in lip position (Fig 3A and S2 Video). Therefore, we model the narrow opening of the bat’s mouth as an array of transmitter elements, each emitting sound originating from the clicking tongue inside the mouth. The elements can be discretely located, representing the gaps between clenched teeth (Fig 3B), or be continuously distributed, representing the narrow gap between parted teeth and lips (S3 Fig panel B). In both scenarios, the array elements are naturally prescribed with a set of relative phase shifts, which vary depending on the distance between the elements and the tongue as well as the sound frequency (Fig 3B). These phase shifts can cause both constructive and destructive sound interference in different directions and produce a strong directivity pattern (Fig 3C) [25]. Importantly, the frequency-dependent phase shifts give rise to the frequency-dependent beam direction changes observed in the experiment.

We implemented this model with boundary element method (BEM) [26] using a head shape obtained from micro computed tomography (μCT) scans of a bat specimen as well as a simplified artificial shape with dimensions comparable to the real head (Fig 3D). In both cases, the model successfully reproduces the beam pattern features shown in the data, including the vertically elongated beam shape, the distinct multi-frequency beam pattern structure, and the uneven azimuthal intensity distribution (Fig 3E–3G, S2 Fig). Specifically, the beam shape is similar between the data and the transmission array model (p > 0.05, Mann-Whitney U-test) but significantly different between the data and the piston model (S7 Fig panel B). The beam centers of the data and the transmission array model exhibit similar frequency-dependent shifts in azimuth, whereas the beam center of the piston model varies only slightly about zero (S7 Fig panel C).

In this model, only array elements on one side of the mouth transmit to produce clicks on the same side (i.e., left array elements produce left-pointing clicks). This is a simplification supported by the observation that each pair of clicks are generated by successive detachment of the two sides of the tongue from bottom of the mouth [17], during which the tongue itself could occlude the sound from one side to the other. Importantly, the experimentally observed beam pattern features cannot be produced by sound transmission from both sides of the mouth (S5 Fig).

**Fig 4:**
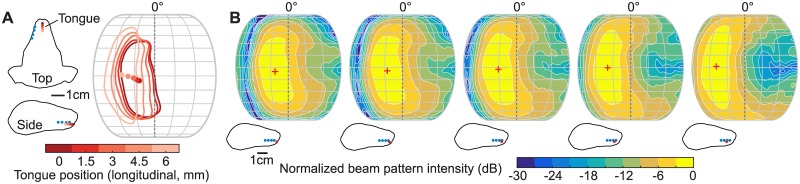
Beam steering using the transmission array model. (A) A series of tongue clicking positions and corresponding −3 dB contours of the normalized model beam patterns (color-coded). Blue dots on the outline of the bat head represent locations of array elements. (B) Model beam patterns at 35 kHz predicted using tongue positions shown in (A). The models were calculated using the bat head mesh derived from μCT scans. The range of azimuth shown is from −90° to 90°. The solid dots inside the contours in (A) and the plus signs (“+”) in (B) indicate the locations of sonar beam center. μCT, micro computed tomography.

To functionally support adaptive inter-click angle changes for accurate target localization, a simple steering mechanism is necessary to realize fine tuning of beam direction at a millisecond time scale [15,18]. Our model provides such flexibility in a biologically-plausible and parsimonious manner: only a single parameter change (tongue clicking location) is required to change beam direction (Fig 4). Here, a 25.7° beam direction shift is induced by a 6-mm shift in tongue location. Importantly, the extent of beam direction changes due to tongue position changes can vary dramatically, depending on the array configuration. For example, a much larger shift in beam direction (44.1°) can be induced by the same tongue position variation simply by moving the array location more forward on the bat’s head (S4 Fig). We note that an important difference between our proposed beam steering mechanism and the so-called “phased array” in the radar and sonar literature is that the phase shifts at array elements are not *individually* controlled in our model. It is the overall phase shift pattern across the array that is varied due to the tongue position change.

In addition, the proposed transmission array model is robust against natural morphological variations. Results of sensitivity analysis suggest that the beam pattern features are generally conserved, irrespective of the head shape and the number and locations of array elements chosen in BEM calculation (Fig 3D–3F, S3 and S4 Figs). This indicates robustness against natural morphological variations, such as those that might occur across individuals or populations.

Our results motivate detailed comparisons between the biosonar employed by the lingual-echolocating Egyptian fruit bat and the more widely-studied laryngeal-echolocating bats. By changing the relative phase shifts of sounds emitted along the parted lips through tongue movements (Fig 3B), the sonar beam steering mechanism of this bat species is in sharp contrast with the all *in-phase* excitation over the gaping mouth described by the conventional piston model. Recall that the piston model is widely used to model the sonar beam of mouth-emitting laryngeal-echolocating species [21]. Instead, it is conceptually analogous to the potential beam steering mechanism postulated for nose-emitting laryngeal-echolocating bats [19,20]. In these studies, simulations were conducted to investigate the extent to which the bats might steer the sonar beam in the azimuthal dimension by manipulating the path differences between the two nostrils to complement their control over vertical beam position via shape modification of the noseleaf. However, unlike the well-documented inter-click sonar beam angle tuning of the Egyptian fruit bat [18], it is not known if nose-emitting laryngeal-echolocating bats are indeed capable of such active manipulation, nor if such beam steering is important for their sonar sensing.

The unusual multi-frequency sonar beam structure of the Egyptian fruit bat raises the question of multi-frequency processing of echo returns. It has been proposed that laryngeal-echolocating bats emitting frequency-modulated (FM) “chirps” may exploit the broad signal bandwidth to improve temporal processing resolution to sub-millisecond level, through a matched-filter-like processing mechanism [27]. The Egyptian fruit bat, which uses broadband but extremely short clicks, may achieve comparable temporal acuity through simple energy detection for returns of highly transient clicks (<100 μs), as has been suggested for dolphins [5]. In addition, based on single-frequency measurements, it has been hypothesized that the sharper drop-off at the inner edges of the Egyptian fruit bat sonar beam enables optimal target localization by “pointing off-axis” using the alternating left- and right-pointing clicks [15]. This hypothesis is in general supported by our observation, since the main lobe across all frequencies overlaps at the inner edges and is as sharp as in the single frequency case (Fig 2B). Furthermore, it has been suggested that the angle-dependent spectral filtering of sonar beam patterns, in which only the region directly in front of the bat’s aim is ensonified with the full signal bandwidth, may help suppress peripheral clutter in FM bats [28]. A similar clutter rejection mechanism may operate in the Egyptian fruit bat as well, since its broadband sonar beam pattern exhibits similar spectral filtering effects. Future investigations into the above questions and whether the paired clicks are processed together or individually will likely provide important insights to the functional link between lingual and laryngeal bat echolocation.

The Egyptian fruit bat belongs to the genus *Rousettus*, whose members are the only lingual echolocating species within the family Pteropodidae [6]. *Rousettus* are frugivorous and rely heavily on visual and olfactory information to forage. They use echolocation as an additional important sensory modality to acquire navigation information in low-light conditions [29]. These factors imply a relatively relaxed constraint on echolocation efficiency compared to the challenges faced by insectivorous bats that need to track highly mobile prey in the dark. Transmission array beam steering and left-/right-pointing target localization strategy of lingual echolocators may enable flexible biosonar behavior in the absence of evolutionary changes in skull morphology, such as those found in laryngeal-echolocating bats [30]. Comparative studies of the hyoid and palate morphology between bats of the genus *Rousettus* and other genera of the same family may provide insights into the evolution of lingual echolocation. A recent study uncovered evidence that at least three other species of Pteropodidae produce clicks with their wings, which supports navigation in the dark [31]. However, sonar-based navigation capability using wing clicks is more limited in comparison to that of *Rousettus* using tongue clicks, and it is thus likely that wing-clicking bats rely largely on visual input. Comparative studies of sonar-guided behaviors under varying light conditions may deepen our understanding of multisensory processing in these animals.

Through measurement and modeling of the sonar beam pattern of lingual-echolocating fruit bats, we present the first evidence that an echolocating animal produces and steers its sonar beam using phase differences passively induced across the side of the mouth (the transmission aperture) by changing location of the clicking tongue. The resultant effects are similar to those of a phased array—an engineering solution widely employed in human-made sonar and radar systems that allows beam direction changes *without* physical modification to the transducer assembly. Additional modeling investigations show that this sonar beam formation mechanism is parsimonious in operation and robust against morphological variations, both of which are essential to successful functioning of biological systems. Our study highlights how engineering principles provide mechanistic insight into the operation of biological systems, and reveals an intriguing parallel between nature and human engineering.

## Materials and methods

### Animals and experimental setup

Three Egyptian fruit bats (*R. aegyptiacus*) were used in the experiment. In each experimental trial, the bat was released by hand from a podium, from which it flew freely through the experimental space (Fig 1A). The space (approximate dimension: 2.3 m × 2.3 m × 2.4 m) was constructed by partitioning a corner of a large room using multiple vertical railings (Unistrut, Atkore International, Harvey, IL). The setup was designed such that the bat’s echolocation and head movement during free flight can be measured at high resolution without complication of landing behavior in spaces of similar size but with solid walls. All walls and railings were covered with acoustic foam and felt to minimize echoes in the acoustic recordings and spurious infrared reflections in the videos. The bats had minimal prior experience with the experiment setup.

The bat’s echolocation clicks were recorded using a 34-element ultrasonic broadband microphone array. The array was composed of 32 electret condenser microphones (the microphones that are normally supplied as part of the D500X Ultrasound Detector/Recorder, Pettersson Elektroniks, Sweden) and 2 precision microphones (40DP 1/8”, G.R.A.S., Denmark and 7016 1/4", ACO Pacific, Belmont, CA). The signals were bandpass-filtered between 10 kHz and 100 kHz using 32 single channel filters (USBPBP-S1, Alligator Technology, CA) and 1 dual channel filter (VBF40, Kemo Filters, Kent, UK). The signals were sampled at 250 kHz through NI PXI-6143 (National Instruments, TX) and 1 MHz through NI PXI-6358 for sounds received by the electret microphones and the precision microphones, respectively.

The bat’s head movements were recorded using a high-speed motion capture system, consisting of 10 motion capture cameras (Vicon T40 and T40S, Oxford, UK) with a sampling rate of 200 frames per second. The motion capture system was calibrated immediately prior to each experimental session. The location and orientation of each microphone were also marked with reflective markers and recorded using this video system. An average positioning error of 3.97 ± 1.93 cm was estimated by comparing the motion capture system measurements with results from time-of-arrival estimates given by speaker playback. In addition, the experiments were monitored by four infrared imaging cameras (Phantom Miro 310, Vision Research, Wayne, NJ).

### Ethics statement

The experimental procedure was approved by the Institutional Animal Care and Use Committee at the Johns Hopkins University (approval number BA14A111). The protocols are in compliance with the Animal Welfare Act regulations and Public Health Service Policy. The university also maintains accreditation by the Association for the Assessment and Accreditation of Laboratory Animal Care International.

### Bat head movement reconstruction

The bat’s head movements while echolocating and flying through the experimental space were reconstructed using reflective markers on the head stage (Fig 1B; attached using Grimas Mastix water-soluble glue). The three-dimensional track of each of the markers was smoothed to reduce jittering errors in the raw motion capture outputs before the head aim was estimated. The bat’s head aim was calculated by the vector pointing from the mid-point of the rear two markers (*b* and *c*) toward the front marker (*a*). The direction pointing upward from the bat’s head (the head normal) was calculated by the cross product $\overset{⃑}{ba}\times \overset{⃑}{ca}$. The head aim, head normal, and their cross product jointly form a bat-centered coordinate system, based on which the beam pattern is calculated (see next section). When clicks were emitted at locations where 1 or more markers were not captured by the motion capture video system, the head aim direction was approximated by the tangent to the trajectory projected onto the x–y plane, and the head normal direction was approximated by the normal vector of the floor plane.

Seventy-six percent of all clicks included in the analysis were projected using head directions derived from the reflective markers. The mean angular error induced by using vectors derived by bat trajectories and room floor was estimated to be 23.7° in the case when only the azimuthal differences are considered, and 36.1° in the case when differences in all three dimensions are considered (S8 Fig) These errors are unlikely to significantly affect our results, because 1) the process of aligning individual clicks according to the center of the −3 dB best-fitting ellipse at 35 kHz (see below) would mitigate errors in bat head directions, and 2) the click directions with respect to the bat head direction is not the focus of analysis in this study, as the majority of our analyses were based on aligning and merging individual click measurements according to the center of the best-fitting ellipse to the −3-dB contour at 35 kHz.

### Individual beam pattern reconstruction

The beam pattern of individual clicks was reconstructed by interpolating the energy spectral density (ESD) of the clicks across the microphones. The ESD measurements were compensated with atmospheric absorption, spherical spreading loss, microphone sensitivity, and microphone receiving directionality prior to interpolation. Radial basis function interpolation was used for the reconstruction, because it incorporates all microphone measurements to derive an expression involving radial basis kernels in predicting each interpolated value [32]. At each click emission, the microphone positions in the “global” coordinate system of the experimental space were transformed into a bat-centered “local” spherical (azimuth-elevation) coordinate system for interpolation. Reporting beam pattern in an azimuth-elevation domain around the bat [23] allows unambiguous comparison with conventional sonar/radar beam pattern measurements. This is different from the projected area approach used in some previous two-dimensional biosonar beam pattern measurements (e.g., [33]).

In addition, five criteria were used to determine the spatial sampling quality of each individual click (S1 Table). Only clicks that satisfy all five criteria were used in the analysis. The beam pattern was reconstructed using a custom-written open-source MATLAB package archived at https://doi.org/10.5281/zenodo.56167.

### Average beam pattern reconstruction

Microphone recordings from all left- and right-pointing clicks were merged to construct average beam patterns (Fig 2A and 2B) to augment observations from individual clicks. Since the bats emit clicks in different azimuthal directions regularly [15,18], an iterative procedure was employed to align the beam axes of all individual clicks before averaging. The alignment was conducted by fitting an ellipse [34] to the −3 dB contour of each individual normalized beam pattern at 35 kHz under the Eckert IV map projection. The azimuth and elevation locations of each microphone were iteratively shifted and rotated until the center of the best-fitting ellipse falls on the map origin (S6 Fig). The average click beam patterns were then constructed by interpolating over the averaged normalized beam intensity values in 10-degree bins across azimuth and elevation. Using Monte Carlo simulations (S1 Text), we showed that the average beam pattern approach is effective in extracting important features under the potential influence of spatial under-sampling (through individual microphones) and measurement noise. In addition, the azimuth and elevation coordinates of the final best-fitting ellipse provide a simple yet quantitative measure of the vertically elongated beam shape. This measure facilitates direct comparison of the beam shape between the experimental data and the models (S7 Fig panel A and B).

### Definition of the center of sonar beam

In this study, the center of the sonar beam (or the “beam center”) at any given frequency is defined as the center of the best-fitting ellipse to the −3 dB normalized beam energy contour at that frequency in the azimuth-elevation domain. This was favored as the definition of the beam center over the location of the microphone that received the maximum sound intensity, because the microphones may under-sample the spatial beam energy distribution, and the maximum intensity microphone location may not reflect the true center of the sonar beam. We support this rationale by verifying that the averaged azimuth-elevation location of all interpolated points with normalized beam energy >− 1 dB is in general very close to the center of the best-fitting ellipse but further away from the maximum intensity microphone location.

### Implementation of the transmission array beam pattern model

The transmission array model was implemented using BEM, which allows the radiation sound field to be computed given a three-dimensional mesh representation of the object boundary and source locations [26]. In this study, the source locations (i.e., elements of the array) are nodes of the bat head mesh located along the mouth opening. To calculate the model beam pattern, the complex sound fields generated by individual array elements are summed coherently [35], with phase shifts determined by the distances between the tongue clicking location within the mouth and the elements *r*_*n*_, i.e.,

$$P_{total}=\sum_{n=1}^{N}P_{n}e^{ikr_{n}},$$ (1)

where *P*_*total*_ is the total pressure field, *P*_*n*_ is the pressure field predicted using the *n*th elements, and *k* is the acoustic wavenumber (*k* = *ω*/*c*, where *ω* is the angular frequency and *c* is the sound speed in air). The computation was implemented using openBEM [26], an open-source MATLAB package available at http://www.openbem.dk.

### Mesh representation of bat head

Two mesh representations were used to implement the transmission array model: the head shape of a *R*. *aegyptiacus* specimen reconstructed using μCT scan and a highly simplified artificial shape scaled to match the dimensions of the scanned bat head. The μCT scans were obtained from a thawed frozen specimen. The head was fixed in 10% formalin and scanned in a Bruker Skyscan 1172 μCT scanner at a resolution of 26 μm (40 kV and 250 amp) with a rotation step of 0.4°. The image stack was reconstructed using NRecon (Bruker, Massachusetts), segmented using Mimics (v.18, Materialise, Belgium), and imported into Geomagic Studio 2014 (3D Systems, South Carolina) for manual cleaning and smoothing. The ears, which were folded midway along their length to accommodate the size of the scanner, were spliced along the fold points, rotated into a natural position based on photographs of live bats, and merged with mesh of the head. The shape was subsequently re-meshed using triangles with an edge length of 0.5 mm. The simplified artificial head shape was constructed using GMSH (http://gmsh.info/) with manually prescribed nodes (S2 Table) connected by straight lines and spherical arcs and scaled to match the length, width, and height dimensions of the scanned bat head.

## Supporting information

S1 Fig Measured beam pattern features of *R*. *aegyptiacus* across multiple frequencies. (A) Normalized beam pattern from the same pair of individual clicks shown in Fig 2. (B) Average clicks from all left- and right-pointing click measurements. Crosses (“x”) indicate projected microphone locations, and plus signs (“+”) indicate the locations of sonar beam center. Other plotting conventions are identical to those in Fig 2A.
(TIF)

S2 Fig Model beam pattern predicted across multiple frequencies. The model was calculated using the bat head mesh derived from μCT scans. The locations of the tongue and array elements were identical to those used in Fig 3. Plus signs (“+”) indicate locations of the center of the beam. μCT, micro computed tomography.
(EPS)

S3 Fig Sensitivity analysis of the transmission array model. (A) Model beam pattern predicted using array elements located more forward (toward the rostrum) compared to those used in Fig 3. (B) Model beam pattern predicted by using a large number of elements forming a continuous array along the narrow opening of the mouth. The models in both (A) and (B) were calculated using the bat head mesh derived from μCT scans. (C) Model beam pattern predicted using the same bat head shape but with the ears removed. All major beam pattern features, including the elongated beam shape, asymmetric intensity distribution, and multi-frequency structure are conserved irrespective of the above model parameter changes. Note that given the same tongue location, the exact locations of the array elements have an effect on the resultant beam direction. Plus signs (“+”) indicate locations of the center of the beam. μCT, micro computed tomography.
(EPS)

S4 Fig Beam steering using a different transmission array configuration. The array elements are located more forward (toward the rostrum) compared to those used in Fig 4. Note the wider azimuthal steering variability compared to that shown in Fig 4. All beam patterns shown are at 35 kHz. Plus signs (“+”) indicate locations of the center of the beam. Other plotting conventions are identical to those in Fig 4.
(EPS)

S5 Fig Model beam pattern predicted using array elements located on both sides of the mouths. Beam patterns were predicted using tongue clicking position in the middle (A) and on the left (B) and right (C) of the midline. The array elements on the left side of the mouth are at the same locations as those shown in Fig 3. Regardless of the tongue position, the two-sided array element arrangement cannot produce beam features as those observed in the experiment.
(EPS)

S6 Fig Procedure of beam pattern alignment. (A) Two examples of the raw measured beam pattern at 35 kHz and corresponding “aligned” results, with the beam axes centered at the origin. Red contours and asterisks show the best-fitting ellipses to the −3 dB contours and the centers of the ellipses.
(EPS)

S7 Fig Data-model comparison of beam shape and beam center locations. (A) Azimuth and elevation extents of ellipses that best fit the −3 dB normalized beam intensity contour at 35 kHz. The comparisons are between experimentally measured individual clicks and Monte Carlo-simulated clicks generated by the transmission array model and the piston model. (B) Distributions of the aspect ratio of best-fitting ellipses shown in (A). The distributions were statistically compared using the Mann-Whitney U-test. (C) Mean azimuthal locations of the center of the beam for experimentally-measured individual clicks and the Monte Carlo-simulated clicks across multiple frequencies. Both the data and the simulated clicks generated using the transmission array model show frequency-dependent beam center shifts, whereas the beam center for simulated clicks generated using the piston model remain near 0°. Note that all individual clicks were aligned according to the center of the best-fitting ellipse at 35 kHz. Therefore, the azimuthal offsets for all frequencies shown here are relative to the azimuthal beam center at 35 kHz (0°).
(EPS)

S8 Fig Angular differences between bat head directions derived from the reflective markers and bat trajectory. (A) Distribution of the angular difference in three dimensions. (B) Distribution of the angular difference in the azimuthal dimension in a bat-centered coordinate. The calculation used the positions of reflective markers and bat trajectories from clicks for which all reflective markers were recorded during experiment.
(EPS)

S9 Fig Average beam pattern constructed based on Monte Carlo-simulated clicks generated using models with randomized beam direction. (A) Five model beam patterns with different beam directions used in the simulation. These model beam patterns are identical to those shown in Fig 4. (B) The average beam pattern constructed based on simulated data generated using the models in (A). The model beam pattern was randomly selected on a click-by-click basis during simulation. Right-pointing clicks were simulated using mirror images of the left-pointing click models. All beam patterns shown are at 35 kHz.
(EPS)

S1 Table Spatial sampling criteria for selecting high quality click measurements. These criteria were designed so that the azimuth-elevation domain covered by the microphone array is sufficiently large, and that the high intensity region of the beam pattern immediately adjacent to the maximum intensity microphone was properly sampled. Here, the maximum intensity microphone refers to the channel that receives the highest sound intensity.
(XLSX)

S2 Table Positions of nodes used to construct the simplified artificial bat head mesh in GMSH. This mesh was subsequently scaled to fit the length, width, and height of the bat head mesh derived from μCT scans. μCT, micro computed tomography.
(XLSX)

S1 Video An example experimental trial. An example trial showing the echolocation beam direction and beam pattern as the bat flew through the experimental space. The video has been slowed down by 16.7 times.
(MP4)

S2 Video Close-up videos of *R*. *aegyptiacus* mouth during echolocation. A compilation of close-up videos showing the relatively small or lack of mouth movement of 3 *R*. *aegyptiacus* during echolocation emission. The video has been slowed down by 20 times.
(MP4)

S1 Text Monte Carlo simulation of beam pattern measurements. (DOCX)

## Abbreviations

BEM: boundary element method
ESD: energy spectral density
FM: frequency-modulated
μCT: micro computed tomography
