# Supplementary figures and images for: Tongue-driven sonar beam steering by a lingual-echolocating fruit bat
Source: PLoS Biol. 2017 Dec 15;15(12):e2003148. doi: 10.1371/journal.pbio.2003148 (PMC5774845; doi:10.1371/journal.pbio.2003148)

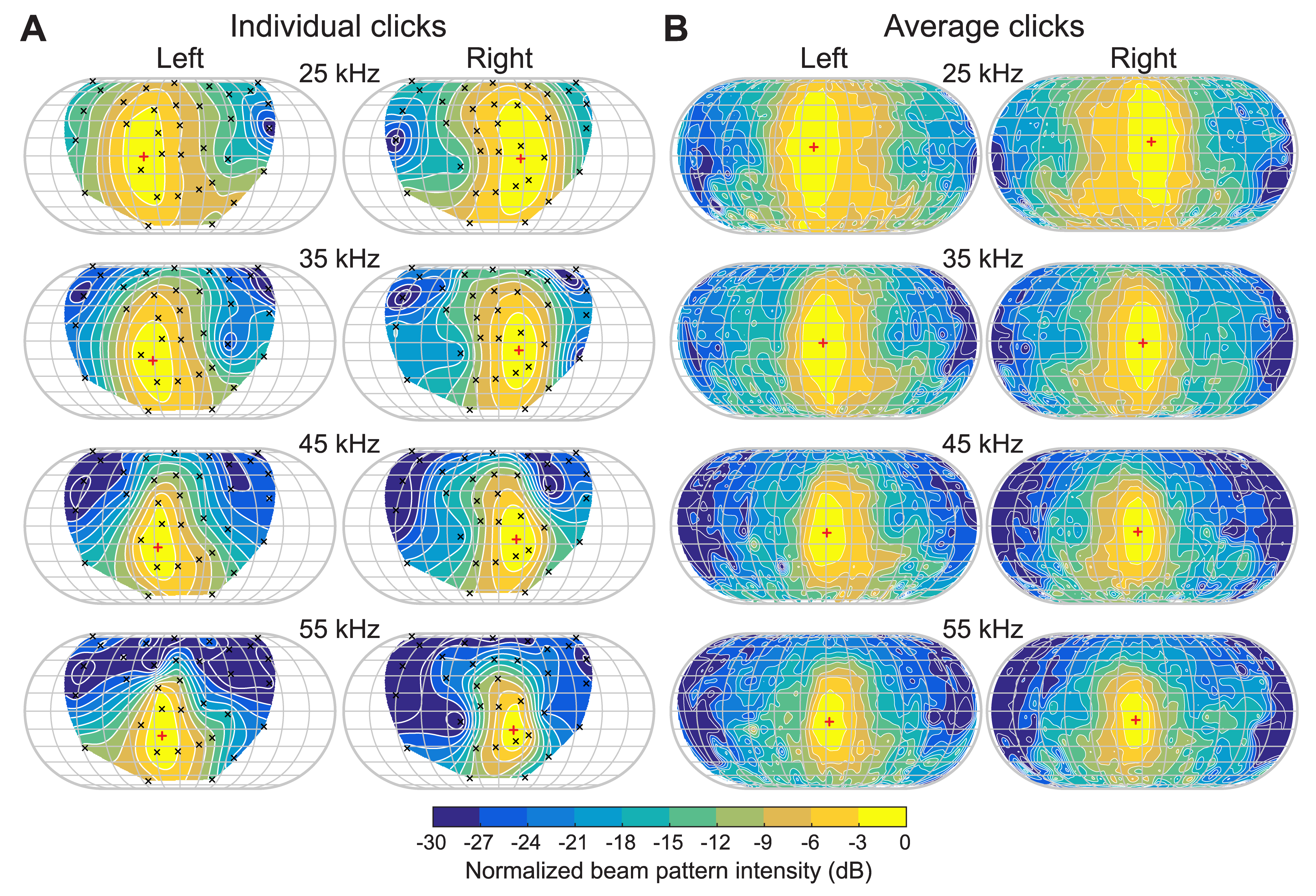

Supplement: S1 Fig — (A) Normalized beam pattern from the same pair of individual clicks shown in Fig 2. (B) Average clicks from all left- and right-pointing click measurements. Crosses (“x”) indicate projected microphone locations, and plus signs (“+”) indicate the locations of sonar beam center. Other plotting conventions are identical to those in Fig 2A. (TIF) [file pbio.2003148.s001.tif]
