# Supplementary material for: Tongue-driven sonar beam steering by a lingual-echolocating fruit bat
Source: PLoS Biol. 2017 Dec 15;15(12):e2003148. doi: 10.1371/journal.pbio.2003148 (PMC5774845; doi:10.1371/journal.pbio.2003148)
Supplement: S1 Text — (DOCX) [file pbio.2003148.s014.docx]

**S1 Text. Monte Carlo simulation of beam pattern measurements**

Monte Carlo simulation was conducted to 1) provide reference data to compare with the experimental data, and 2) verify the reliability of beam pattern reconstruction procedure in extracting important beam pattern features. Two beam pattern models were used in the simulation, including the conventional circular piston model [21] and the transmission array model proposed in this study. The simulations used the bat and microphone locations recorded during the experiment. For each click emission, the model beam pattern is projected from the bat position toward all microphones in the bat-centered “local” coordinate system, with the beam axis pointing to the center of the sonar beam (see definition in Materials and Methods) derived from experimental measurements. Gaussian-distributed noise with zero mean and a standard deviation of 1 dB was subsequently added in an ad-hoc manner in the logarithmic scale to produce simulated beam pattern measurements.

Monte Carlo simulation conducted using the piston model showed that the measured elongated beam shape is not an artifact of spatial sampling bias. The model beam pattern was generated using a piston aperture radius of 4.91 mm, determined by averaging the -3 dB beamwidths in both azimuth and elevation of the experimental data. The simulation showed that the beam pattern aspect ratio (ratio of the elevation and azimuth values of the best-fitting ellipse) of the circular piston is significantly different from those of the data (*p*<0.05, Mann-Whitney U-test; S7 Fig panel A and B), indicating that the observed elongated beam shape does not result from sampling artifact.

Monte Carlo simulation conducted using the transmission array model showed that the average beam pattern reconstruction procedure is reliable in extracting important beam features by pooling multiple click measurements. Two sets of simulations were conducted. In the first set, the two fixed model beam patterns shown in Fig 3E were used to generate all clicks. In the second set, one of the five model beam patterns shown in Fig 4 was randomly selected to generate simulation at each click transmission. The latter set was designed to simulate natural variability among clicks. The resultant average beam patterns (Fig 3F and S9 Fig for the first and second set, respectively) preserved the beam pattern features shown in the model, indicating the reliability of the average beam pattern reconstruction procedure. In addition, the distribution of the beam pattern aspect ratio and the frequency-dependent beam center shifts are similar to those of the measured clicks (S7 Fig), and the simulated average beam patterns show great qualitative resemblance to the experimental data (Fig 2). These demonstrate the power of the transmission array model.
